# The Immune Microenvironment Landscape of Pituitary NeuroEndocrine Tumors, a Transcriptomic Approach

**DOI:** 10.3390/genes15050531

**Published:** 2024-04-24

**Authors:** Sandra Vela-Patiño, Ma. Isabel Salazar, Keiko Taniguchi-Ponciano, Eduardo Vadillo, Erick Gomez-Apo, Aurea Escobar-España, Vadim Perez-Koldenkova, Laura Bonifaz, Cristina Aguilar-Flores, Daniel Marrero-Rodríguez, Moises Mercado

**Affiliations:** 1 Unidad de Investigación Médica en Enfermedades Endocrinas, Hospital de Especialidades, Centro Médico Nacional Siglo XXI, Instituto Mexicano del Seguro Social, Av. Cuauhtémoc 330, Col. Doctores, Ciudad de México 06720, Mexicokeiko.taniguchi@hotmail.com (K.T.-P.); 2 Laboratorio Nacional de Vacunología y Virus Tropicales, Departamento de Microbiología, Escuela Nacional de Ciencias Biológicas, Instituto Politécnico Nacional, Ciudad de México 11350, Mexico; 3 Unidad de Investigación Médica en Enfermedades Oncológicas, Hospital de Oncología, Centro Médico Nacional Siglo XXI, Instituto Mexicano del Seguro Social, Av. Cuauhtémoc 330, Col. Doctores, Ciudad de México 06720, Mexico; 4 Área de Neuropatología, Servicio de Anatomía Patológica, Hospital General de México “Dr. Eduardo Liceaga”, Ciudad de México 06720, Mexico; erickapo@hotmail.com (E.G.-A.);; 5 Laboratorio Nacional de Microscopia Avanzada, Centro Médico Nacional Siglo XXI, Instituto Mexicano del Seguro Social, Av. Cuauhtémoc 330, Col. Doctores, Ciudad de México 06720, Mexico; 6 Unidad de Investigación Médica en Inmunoquímica, Hospital de Especialidades, Centro Médico Nacional Siglo XXI, Instituto Mexicano del Seguro Social, Av. Cuauhtémoc 330, Col. Doctores, Ciudad de México 06720, Mexico; 7 Coordinación de Investigación en Salud, Centro Médico Nacional Siglo XXI, Instituto Mexicano del Seguro Social, Av. Cuauhtémoc 330, Col. Doctores, Ciudad de México 06720, Mexico; 8 Unidad de Investigación Médica en Inmunología, Hospital de Pediatría, Centro Médico Nacional Siglo XXI, Instituto Mexicano del Seguro Social, Av. Cuauhtémoc 330, Col. Doctores, Ciudad de México 06720, Mexico; 9 Centro de Cancer, Hospital American British Cowdray, Sur 136 116, Las Américas, Álvaro Obregón, Ciudad de México 01120, Mexico

**Keywords:** immune microenvironment, pituitary tumors, cytokines, chemokines, M2 macrophages

## Abstract

Pituitary neuroendocrine tumors (PitNET) are known to be variably infiltrated by different immune cells. Nonetheless, their role in pituitary oncogenesis has only begun to be unveiled. The immune microenvironment could determine the biological and clinical behavior of a neoplasm and may have prognostic implications. To evaluate the expression of immune-related genes and to correlate such expression with the presence of infiltrating immune cells in forty-two PitNETs of different lineages, we performed whole transcriptome analysis and RT-qPCR. Deconvolution analysis was carried out to infer the immune cell types present in each tumor and the presence of immune cells was confirmed by immunofluorescence. We found characteristic expression profiles of immune-related genes including those encoding interleukins and chemokines for each tumor lineage. Genes such as *IL4-I1*, *IL-36A*, *TIRAP*, *IL-17REL*, and *CCL5* were upregulated in all PitNETS, whereas *IL34*, *IL20RA*, and *IL-2RB* characterize the *NR5A1-*, *TBX19*-, and *POU1F1*-derived tumors, respectively. Transcriptome deconvolution analysis showed that M2 macrophages, CD4+ T cells, CD8+ T cells, NK cells, and neutrophils can potentially infiltrate PitNET. Furthermore, CD4+ and CD8+ T cells and NK cells infiltration was validated by immunofluorescence. Expression of *CCL18, IL-5RA*, and *HLA-B* as well as macrophage tumor infiltration could identify patients who can potentially benefit from treatment with immune checkpoint inhibitors.

## 1. Introduction

Pituitary neuroendocrine tumors (PitNET) represent the second most frequent intracranial neoplasia after meningiomas. They are clinically classified as clinically functioning and non-functioning tumors [1]. Clinically functioning tumors result in hormonal hypersecretion syndromes: GH-secreting PitNET or somatotrophinomas causing acromegaly/giantism; PRL-secreting PitNET, giving rise to galactorrhea, amenorrhea, and infertility; the rare TSH-PitNET causing central hyperthyroidism; and ACTH-secreting PitNET, which are the cause of Cushing’s disease [1]. Clinically non-functioning PitNET (CNF PitNET) do not result in a hormonal hypersecretion syndrome, although over half of them are of gonadotroph differentiation since they immunostain for LH or FSH β-subunits as well as for α-subunit [1]. CNF PitNET also comprise silent GH, PRL, and ACTH tumors as well as null-cell tumors, which do not immunostain for any hormone or transcription factor. TSH, PRL, and GH-secreting PitNET derive from the same cell precursor and immunostain for the transcription factor POU1F1, whereas ACTH- and gonadotropin-secreting PitNET immunostain for the transcription factors TBX19 and NR5A1, respectively [1].

It is now known that the immune system plays an important role in the development and progression of different types of benign and malignant neoplasms [2]. These actions of the immune system include the promotion of an inflammatory microenvironment, which contributes to genomic instability and epigenetic modifications that translate into an increased cell proliferation, angiogenesis, and activation of anti-apoptotic pathways [2]. A significant proportion of PitNET have been found to have varying degrees and types of immune cell infiltration [3,4,5,6]. Functioning PitNET have three to four times more macrophage infiltration than non-tumoral pituitary tissue [7]. B-lymphocytes and natural killer cells (NK) are also known to infiltrate PitNET [8]. Immune cells within the PitNET microenvironment can potentially release different cytokines and chemokines, which can modify the biological behavior of these neoplasms [7]. Immune checkpoint inhibitors (ICi) have been used in a handful of aggressive pituitary neuroendocrine tumors with reasonable success [9,10,11]. In this context, and based on their immunological profile and the expression of different immune checkpoint molecules, PitNET have recently been classified into tumors sensitive to anti-CTLA-4 monoclonal antibodies, tumors sensitive to anti-PD-L1 monoclonal antibodies, and tumors resistant to both anti-CTLA-4 and anti-PD-L1 antibodies [6]

In the present study we evaluated the immunological microenvironment of PitNET using a transcriptomic approach that allowed us to infer the types of infiltrating immune cell populations by whole transcriptome cellular deconvolution analysis. Such findings were also validated by immunofluorescence in some representative samples. We developed a novel immune score that would aid in identifying PitNET patients who would be likely to benefit from treatment with ICi.

## 2. Materials and Methods

### 2.1. Patients and Tissue Samples

Forty-two PitNET samples were collected, including twenty CNF PitNET (fourteen gonadotrope, three null, cell and three silent ACTH tumors), ten GH-secreting PitNET, six ACTH-secreting PitNET, four TSH-secreting PitNET, and two prolactinomas. All tumors were obtained from patients diagnosed, treated, and undergoing follow up at the Endocrinology Service and the Neurosurgical department of Hospital de Especialidades, Centro Médico Nacional Siglo XXI of the Instituto Mexicano del Seguro Social from May 2016 to May 2019. All samples were from treatment naïve patients who had not received radiation therapy or any other pharmacological intervention prior to surgery. Six non-tumoral pituitary glands were obtained within 10 h of death from autopsies performed at the Pathology Department of Hospital General de México and were used as controls. All participating patients were recruited with signed informed consent and ethical approval from the Comisión Nacional de Ética e Investigación Científica del Instituto Mexicano del Seguro Social in accordance with the Helsinki declaration as previously reported [12,13].

### 2.2. RNA Purification

Total RNA was extracted from PitNET and non-tumoral pituitaries using the miRNAeasy Mini Kit (Qiagen Inc., Redwood City, CA, USA) according to manufacturer’s instructions. Tissue samples were disrupted and homogenized in 700 μL Qiazol Lysis Reagent. They were then incubated at room temperature for 5 min. Next, 200 μL of chloroform was added, and samples were incubated at room temperature for 3 min. The mixture was centrifuged at 12,500 rpm for 15 min at 4 °C. The aqueous phase was transferred to a fresh tube and mixed with an equal volume of 70% ethanol. Samples were then transferred to an RNAeasy Column in a 2 mL tube and centrifuged at 10,000 rpm for 15 s. After centrifugation, 700 μL of RW1 buffer was added and the mixture was centrifuged at 10,000 rpm for 15 s. Flow-through was discarded and 500 μL of RPE buffer was added to the membrane and then centrifuged at 10,000 rpm for 15 s (2×). The column was transferred to a new collection tube adding 30 μL of RNAse free water and centrifuged for 1 min at 10,000 rpm. RNA was quantified using a Nanodrop-ND-1000 spectrophotometer (Thermo Scientific, Wilmington, DE, USA); RNA integrity was evaluated by Bioanalyzer 2100 as previously reported [12,13].

### 2.3. Microarray GeneChip Clariom D Assay

The microarray used for these studies was Affymetrix Clariom D, which allows us to analyze whole coding transcriptome at the gene and exon level as well as non-coding RNA such as lincRNA, miRNA, and circRNA. Sample amplification and preparation for microarray hybridization was performed according to Affymetrix specifications. Briefly, 100 ng of total RNA was reversely transcribed into cDNA, amplified by in vitro transcription, and reversely transcribed to cDNA again. Fragments between 40 and 70 bp were generated enzymatically, labelled, and hybridized onto the microarray chips in an Affymetrix hybridization oven at 60 rpm and 45 °C for 17 h. Chips were washed according to the stablished protocols (Affymetrix, Santa Clara, CA, USA) with a GeneChip fluidics station 450, and finally scanned with an Affymetrix 7G GeneChip scanner. The raw data (CEL files) has been uploaded into the Gene Expression Omnibus (GEO), which is hosted by the National Center for Biotechnology Information (NCBI) under the accession number GSE147786. As previously reported [12,13].

### 2.4. Bioinformatic Analysis of PitNET Transcriptome

A total of 6 control and 42 PitNET experiments were analyzed, including 2 technical replicates. Data sets were analyzed by means of CEL files with the Expression Console, Partek Genomics Suite 7.19v software (Partek Incorporated, Saint Louis, MO, USA), and the Transcriptome Analysis Console (Affymetrix, Santa Clara, CA, USA). Pearson and Spearman correlations were performed, and probe sets were summarized by means of Median Polish and normalized by quantiles with no probe sets excluded from the analysis. Background noise correction was achieved by means of Robust Multi-chip Average (RMA) and data were log^2^ transformed. Data grouping and categorization was achieved by principal component analysis (PCA). Differentially expressed genes were determined by means of ANOVA. Gene expression was considered to be altered upon identifying a +2 or −2-fold change compared to non-tumoral pituitaries, *p* ≤ 0.05 and FDR ≤ 0.05 parameters, as previously reported [12,13].

### 2.5. Deconvolution Analysis for Immune Cell Infiltration Inference

Deconvolution analysis was carried out using four different algorithms, namely ImmuCellAI, CIBERSORT, TIMER, and EPIC. However, we used the CIBERSORT algorithm in the description of the results because it better represents our transcriptome data and better correlates with the immunofluorescence results. CIBERSORT is an online tool (Cell Type Identification by Estimating Relative Samples Of RNA Transcripts) that solves the equation m = f × B, where m is a mixture of mRNA, f is a vector that denotes the cell fractions that make up the mixture, and B is an array of gene expression profiles characteristic of each cell subtype. CIBERSORT is based on the application of v-SVR (nu-support vector regression) where limits of a hyperplane are determined that fits most of the possible points at a constant distance and where a regression is performed. Support vectors are genes selected from B. CIBERSORT was used for the present work with the default parameters, using the LM22 expression profile matrix (22 types of immune cells) and 100 permutations to calculate the *p*-value associated by Monte Carlo Sampling. The mRNA mixture used was a matrix constructed with the expression values of each sample obtained by microarrays, as previously reported [13].

### 2.6. Identification of Determinants of Tumor Immunogenicity and Prediction of Response to ICi 

To identify the potential response or non-response to immunotherapies, we used immunophenogram by The Cancer Immune Atlas. The immunophenogram enables the calculation of an aggregated score—immunophenoscore—based on the expression of major determinants, identified by a random forest approach. The immunophenscore (IPS) was calculated on a 0-10 scale based on the expression of the representative genes or gene sets of the immunophenogram. Samplewise z-scores are positively weighted according to stimulatory factors (cell types) and negatively weighted according to inhibitory factors (cell types) and averaged (https://tcia.at/tools/toolsMain accessed on 7 January 2024).

### 2.7. Immunofluorescence Assays and Confocal Microscopy

Paraffin-embedded, formalin-fixed tissue blocks were cut and the sections stained with hematoxylin-eosin and reviewed by a pathologist. Sections (3 μm) were cut and placed onto coated slides. Remaining paraffin in the slides was removed at 70 °C for 40 min. A train of solvents (Xylol/Ethanol) was used for rehydration of the tissues. Heat-induced antigen retrieval was performed (citrate buffer pH 6.0, sodium citrate 10 μM) at 120 °C for 20 min. Tissue was permeabilized for 2 h (10 mg/mL bovine serum albumin, 5% horse serum, 0.02% sodium azide, and 0.5% Triton). Permeabilized tissue was incubated for 18 h with primary antibodies anti-CD8 dilution 5 µm/mL (eBiosciences, San Diego, CA, USA, #cat 53-0008-80), anti-CD4 dilution 5 um/mL (Sigma, Livonia, Michigan, USA, Darm, Ger #cat HPA004252-100UL), anti-CD66B dilution 1:50 (Thermo Fisher, Pleasanton, CA, USA #cat MA1-26144), anti-CD11c dilution 1:50 (abcam, #cat ab52632), anti-HLA-DR dilution 1:100 (Thermo Fisher, Pleasanton, CA, USA #cat YD1), anti-BDCA3 dilution 1:50 (abcam, #cat ab6980), anti-NKP46 dilution 1:50, anti-IFN-ɣ dilution 20 µm/mL (R&D systems, Minneapolis, MN, USA, #cat MAB285-100), anti-CD56 dilution 15 µm/mL (R&D systems, Minneapolis, MN, USA, #cat AF2408), and anti-IL-17 dilution 1:100 (R&D systems, #cat AF-317-NA). Incubation of secondary antibodies anti-goat AF594, anti-rabbit AF647, anti-rabbit 594, and anti-mouse AF488 (Jackson Immunoresearch, West Grove, PA, USA) was performed for 2h. Nuclei were stained with Hoechst (Invitrogen, Carlsbad, CA, USA) for 10 min. Finally, tissues were mounted with Vectashield (Vector Laboratories, Newark, CA, USA). Images were obtained on a Nikon Ti Eclipse inverted confocal microscope (Nikon Corporation, Tokio, Japan) using NIS Elements v.4.50. Imaging was performed using a 20× (dry, NA 0.8) objective lens. Zoom was performed at 3.4×. Images were analyzed using FIJI ImageJ Software V1.54.

### 2.8. Reverse Transcription and qPCR

After purification, 1 μg of total RNA was retrotranscribed in a 20 μL final volume reaction with the SuperScript VILO Master Mix (Applied Biosystems, Foster City, CA, USA), 4 μL of Master Mix were added, and the reaction mixture was incubated at 25 °C for 10 min, 42 °C for 60 min, and 85 °C for 5 min, according to manufacturer protocols. For RT-qPCR of *IL-34* (Hs01050926_m1), *IL-20RA* (Hs01011609_m1), and *IL-2RB* (Hs01081697_m1), all reagents were purchased from Applied Biosystems (Foster City, CA, USA), and conditions were as follows: 10 μL of Taqman Universal Master Mix II, 1 μL of each Taqman probe, 200 ng of cDNA in a 20 μL final volume, according to manufacturer’s recommendation. *RPLP0* (Hs99999902_m1) was used as endogenous control and all reactions were performed in triplicate in the Step one thermal cycler (Applied Biosystems, Foster City, CA, USA). 2^−ΔΔCt^ relative expression was calculated, as previously reported [12,13]. 

### 2.9. Statistical Analysis

The Mann–Whitney U test was used for pairwise group comparisons of non-normally distributed variables. One-way analysis of variance and Kruskal–Wallis test was used for comparisons between more than two groups. A *p*-value < 0.05 was considered statistically significant. All analyses were carried out using version 7 of GraphPad Prism. 

## 3. Results

### 3.1. Immune Response Gene Profiling in Pituitary Neuroendocrine Tumor

**Figure 1:**
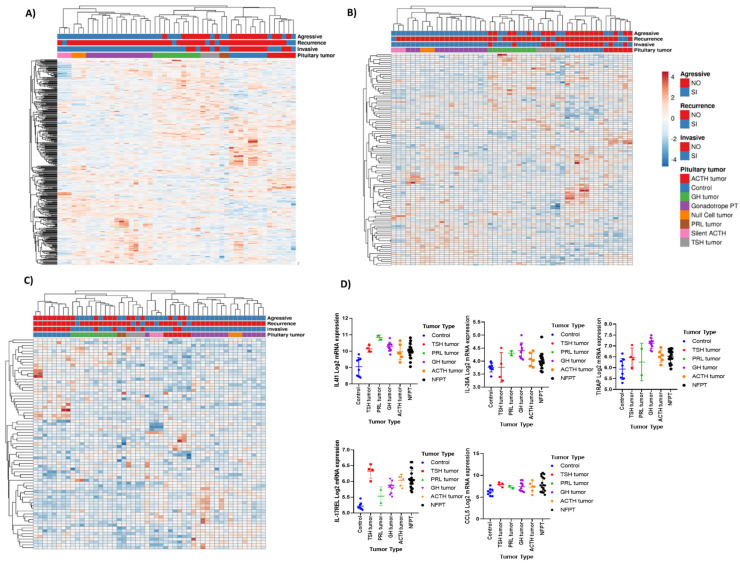
Immune related gene expression in pituitary tumors. (**A**) Expression of filtered genes related to the immune response, such as chemokines and interleukins and their receptors, TNFs, growth factors, interferons, HLAs, and complement proteins. Expression profiles were identified for each type of pitNET. (**B**) Hierarchical cluster of genes related to interleukins and interleukin receptors, presenting characteristic transcription factor expression profiles. (**C**) Hierarchical cluster of genes related to chemokines and their receptors. On the *x*-axis, pit-NET are grouped according to the WHO classification, including null cell tumors, corticotropinomas, prolactinomas, silent corticotropinomas, somatotropinomas, thyrotropinomas, and gonadotropinomas, and they are also clustered by clinical characteristics such as invasiveness, recurrence, or aggressiveness; gene expression is depicted on the *y*-axis. (**D**) Expression of immune response genes that were upregulated in all pitNET; the *x*-axis depicts the type of pituitary pitNET types and the *y*-axis the log2 mRNA relative expression. The image was created using Clustvis (https://biit.cs.ut.ee/clustvis/) (accessed on 7 January 2024) and GraphPad version 7 based on data analyzed by TAC.

From the complete transcriptome of PitNET, filtering was performed considering genes related to immune response. The selected genes of the immunological response include complement proteins, HLA molecules, growth factors, interleukins (IL), chemokines, and their respective receptors, as well as members of the tumor necrosis factor family (TNF’s). A total of 462 genes were differentially expressed in tumors compared to non-tumoral control glands; 220 were up-regulated and 242 were down-regulated. Interestingly, the expression of immune related genes can readily distinguish tumor from nontumoral gland. This immune gene signature segregates along each tumor lineage and the transcription factor that determines their terminal differentiation: POU1F1-dependent GH-, TSH-, PRL-secreting PitNET; NR5A-1-driven gonadotrophinomas; and TBX19-dependent ACTH-secreting PitNET (Figure 1A). The differential expression of 108 genes, particularly including those encoding ILs and their receptors, defined a distinctive profile for each PitNET type (Figure 1B). The differential expression profiles of genes encoding chemokines and their receptors also showed a tendency to distinguish each PitNET lineage, although this was not as evident as with the expression of ILs and their receptors. 

**Figure 2:**
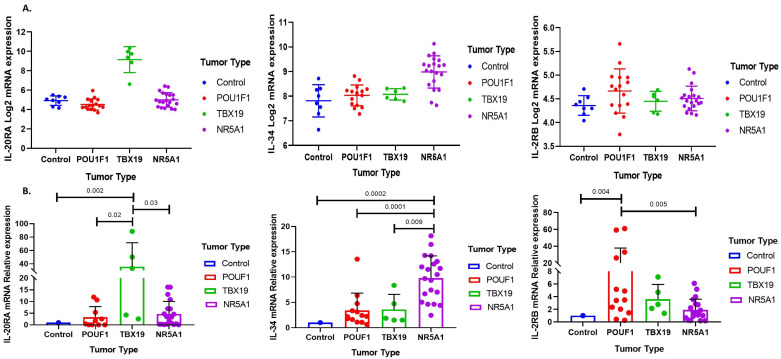
Characteristic immune-related gene expression in pitNET. (**A**) Immune response genes filtered for each pitNET lineage. IL20RA, IL-34, and IL2RB were chosen for TBX19-, NR5A1-, and POU1F1-derived pitNET, respectively. The expression values shown were obtained from whole transcriptome data. The *y*-axis depicts the log2 mRNA expression, and the *x*-axis, the pitNET tumor type. (**B**) Validation of the selected genes characteristic of each pitNET lineage using real-time PCR; the *y*-axis represents the relative mRNA expression and the *x*-axis, the type of tumor. Statistical comparison was performed using a Mann–Whitney U test. All *p* values < 0.05 were considered statistically significant. The image was generated using the GraphPad Prism 7 program.

Genes encoding molecules such as *IL-4I1* (IL4-induced-1), *IL-36A*, *TIRAP* (toll-IL-1 receptor adaptor protein), *IL-17REL*, and *CCL5* (C-C motif chemokine ligand 5) were upregulated in all PT (Figure 1D). NR5A1-derived CNF PitNET expressed the genes encoding *IL-34*, whereas *IL-20RA* and *IL-2RB* were distinctly expressed by TBX19-derived corticotrophic tumors and POU1F1-dependent GH-, TSH-, and PRL-secreting PitNET, respectively. We corroborated our microarray transcriptomic findings by RT-qPCR (Figure 2). 

### 3.2. Deconvolution Analysis for Identification of Immune Cellular Infiltrates in PitNET

**Figure 3:**
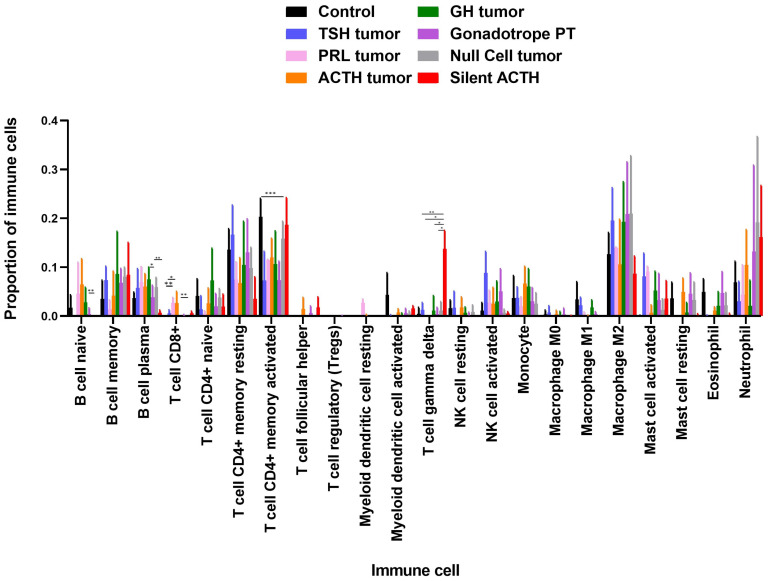
Immune cells potentially infiltrating PitNET. Deconvolution analysis depicting the proportions of immune response cells potentially infiltrating pitNET. The *y*-axis depicts the proportions of cells in the tumor immune microenvironment, while the *x*-axis represents the type of immune cells analyzed by the CIBERSORT algorithm. The analysis reveals a higher proportion of infiltrating immune cells in pitNET compared to non-tumoral pituitary tissue. These include CD4 +T cells, CD8 +T cells, NK cells, M2- macrophages, and neutrophils. Statistical analysis was conducted using the Mann–Whitney U test, a *p* value < 0.05 was considered statistically significant; *p* < 0.05 (*), *p* < 0.01 (**), *p* < 0.001 (***). The image was generated using the GraphPad Prism 7 program.

**Figure 4:**
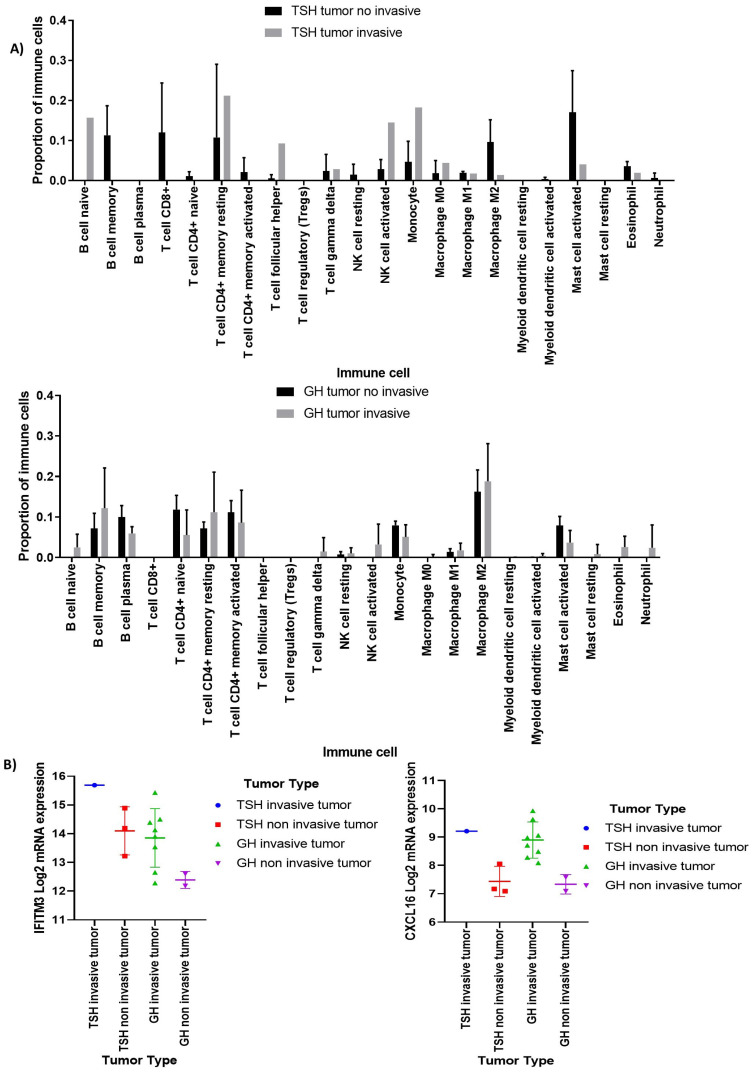
Immune infiltration estimates according to invasiveness. Deconvolution analysis displaying the proportion of infiltrating immune response cells in invasive pituitary tumors. (**A**) In invasive somatotropinomas, a higher proportion of neutrophils, eosinophils, and mast cells was observed, while in invasive thyrotropinomas, a higher proportion of monocytes and NK cells was noted. The *y*-axis depicts the cell proportion in the tumor immune microenvironment and the *x*-axis represents the immune cells analyzed by the CIBERSORT algorithm. (**B**) Differentially expressed genes in invasive tumors, such as the chemokine CXCL16 and IFITM3, are shown. The expression values presented were obtained from whole transcriptome data. The *y*-axis represents the log2 mRNA expression and the *x*-axis the type of pitNET.

Deconvolution analysis was performed using the complete transcriptome of PitNET by means of the CIBERSORT algorithm, which estimates the proportions of 22 types of immune cells. Beside immune response cells, we also identified other cells present in the tumor such as endothelial cells and cancer associated fibroblasts. Tumor cells comprised the vast majority of cell populations; immune response cells were found infiltrating the tumor as well as endothelial cells, but no cancer associated fibroblast were predicted (Supplementary Figure S1). Immune cell populations potentially infiltrating PitNET include CD4+ T cells, CD8+ T cells, M2 macrophages, neutrophils, and NK cells (Figure 3). Once we identified the immune cell populations that with greater abundance infiltrate the different types of PitNET, we sought clinical-pathological correlations between the types of infiltrating cells and tumor characteristics such as invasiveness, recurrence, and aggressiveness. Only two PitNET subtypes, invasive somatotrophinomas and thyrotrophinomas, showed a potential correlation between invasiveness and immune cell infiltration according to our deconvolution analysis (Figure 4A). Invasive GH-secreting PitNET were found to have increased numbers of infiltrating myeloid cell lineages such as neutrophils, eosinophils, and mast cells. Invasive TSH-secreting PitNET had immune cell infiltrates with a predominance of monocytes, CD4+ T cells, and NK cells. 

Clinical and biological features such as cavernous sinus invasion in GH- and TSH-secreting PitNET was associated with upregulation of the genes encoding *CXCL16* (C-X-C-motif chemokine ligand 16) and *IFITM3* (interferon-induced transmembrane protein 3) (Figure 4B).

### 3.3. Identification of Immune Cell Infiltrates by Immunofluorescence

**Figure 5:**
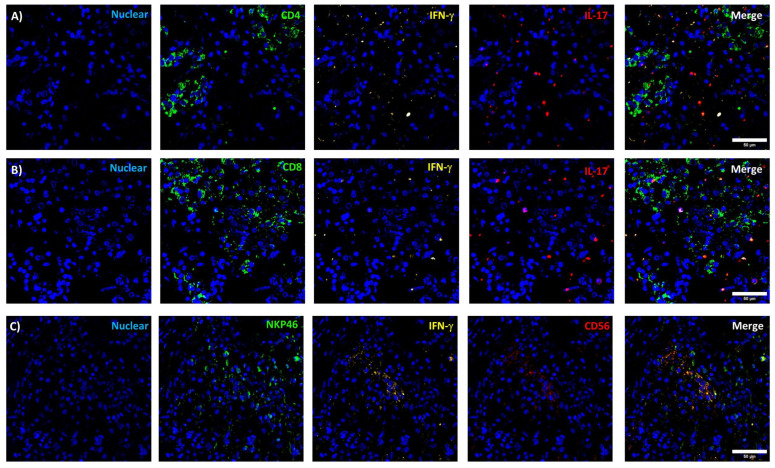
Immune cell infiltrates in pitNET. Specific immune cells visualized by immunofluorescence using antibodies against CD4, IFN-ɣ, CD8, IL-17, NKP46, and CD56. CD4+ T, CD8+ T cells, and NK cells are depicted in panels (**A**), (**B**), and (**C**), respectively.

To corroborate the deconvolution analysis data, immunofluorescence was performed in a subset of 12 samples: 3 CNF PitNET, 3 somatotropinomas, 2 corticotropinomas, 2 thyrotopinomas, and 2 prolactinomas. CD4+ and CD8+ T lymphocytes as well as NK cells were found infiltrating all these tumor types, albeit in different proportions. IFN and IL-17 expression was sought in order to ascertain the activation status of the infiltrating immune cells and to differentiate between Th1 or Th17 cells. Interestingly, CD4+, CD8+ T cells, and NK cells were found to produce small amounts of IFNγ but IL-17 was expressed by the tumoral cells themselves (Figure 5).

### 3.4. Immunophenoscore Predicts Potential Response to Immunotherapy

**Figure 6:**
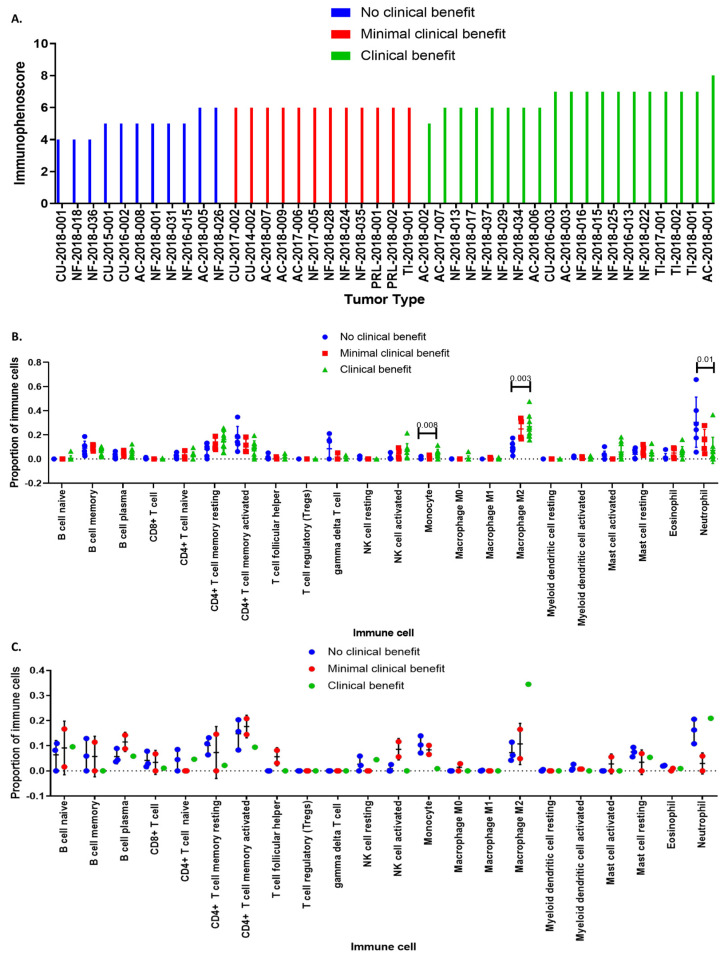
Prediction of response to ICi in PitNET based on the expression of CTLA-4 and PD-L1. (**A**) Immunophenoscore results in the different samples. (**B**) Differences in immune cell infiltrates between CNF PitNET that may benefit from ICi from those who may not; M2 macrophage infiltration was associated with potential clinical benefit. (**C**) Differences in cell populations between responder and non-responder ACTH-secreting tumor. AC = GH-secreting PitNET, CU = ACTH-secreting PitNET, NF = LH/FSH-secreting PitNET, PRL = PRL-secreting PitNET, TI = TSH-secreting PitNET. Green color depicts all tumors that could have benefited from ICi treatment, red color depicts all tumors that could have a minimal benefit from ICi treatment, and blue color depicts all tumors that do not have any benefit from ICi treatment. Figures obtained using the GraphPad Prism 7 program.

**Figure 7:**
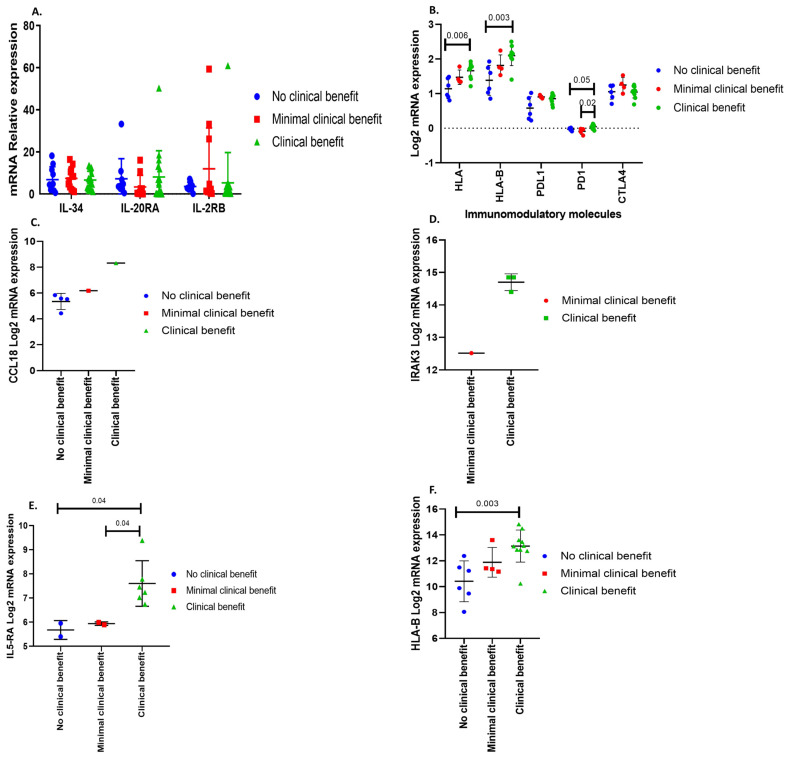
(**A**) Differential expression of genes encoding IL-34, IL-20RA, and IL-2RB among no clinical benefit, minimal benefit, and clinically significant benefit from ICi. (**B**) Upregulation of HLA, HLA-B and PD-1 in CNF pitNET. (**D**) Genes that could help us identify PitNET that may clinically benefit from treatment with ICi: (**C**) CCL18 for ACTH-secreting tumors, (**D**) IRAK3 in TSH-secreting PitNET, (**E**) IL-5RA for GH-secreting PitNET and (**F**) HLA-B for CNF PitNET. Green color depicts all tumors that could have benefited from ICi treatment, red color depicts all tumors that could have minimal benefit from ICi treatment, and blue color depicts all tumors that do not have any benefit from ICi treatment. Figures obtained using the GraphPad Prism 7 program.

Transcriptome data was loaded into the The Cancer Immune Atlas immunophenogram algorithm. Tumors were segregated into three different groups according to the potential response to immunotherapy: (1) no clinical benefit, (2) minimal benefit or clinically not significant benefit, and (3) clear clinical benefit (Figure 6A). This classification was based on the cellular composition of the intratumoral immune infiltrates, the gene expression level of *CTLA-4*, *PD-1*, *PD-L1*, cancer antigenomes comprising neoantigens, tumor heterogeneity, and predetermined sets of genes like HLA molecules, immunostimulators, and immunoinhibitors (Figure 7B) (12–14). This segregation takes place regardless of the type of hormone secreting or non-secreting tumor or its cell lineage.

All PitNET types expressed the three immunotherapy response genes, which underscores the importance of analyzing each tumor individually in a precision medicine approach. Once the response groups were identified, transcriptome-wide analysis was carried out to identify genes related to immune response. *CCL18* (C-C motif chemokine ligand 18) was identified in ACTH-secreting tumors (Figure 7C)*, IRAK3* in TSH-secreting tumors (Figure 7D), *IL-5RA* in GH-secreting PitNET (Figure 7E), and *HLA-B* in CNF PitNET (Figure 7F). Previously selected genes, characteristic for each lineage (*IL-34, IL-20RA*, and *IL2-RB*), were tested to look for a correlation with the response to therapy with ICi; no statistical differences were found between the groups (Figure 7A).

We also performed deconvolution analysis using CIBERSORT to unravel the immunological profiles that characterize responsiveness to treatment with ICi. Patients harboring CNFPT and ACTH-secreting PitNET in whom immunotherapy resulted in a clinical benefit tended to have tumors with a notorious infiltration of M2-macrophages (Figure 6B). Tumors from non-responding patients lacked M2-macrophage infiltration (Figure 6C).

## 4. Discussion

In the present study we have characterized the PitNET immune microenvironment landscape. We identified cells that commonly infiltrate PitNET by means of a deconvolutional analysis derived from extensive transcriptomic information, as well as by immunofluorescence. We also documented the immune-related gene expression of pituitary tissues finding potentially distinctive profiles for each tumor lineage. With this analysis, we aimed to integrate the immune cell landscape with tumor cell gene expression analysis. We have identified the cytokine and chemokine alongside with other immune-related gene expression to clarify the differences between tumor microenvironment and how it could attract and/or interact immune cells in each cell lineage. This could help to understand the different mechanisms by which pituitary tumors attract the immune cells since cytokines and chemokines are key modulators of inflammation and are produced primarily by cells to recruit leukocytes to the site of injury [14]. Better understanding of the immune-tumor cell interaction could help to develop new immune based therapies. It could also help to predict which patients could have better response in the case of use of immune based therapies as we apply the prediction algorithm and complement it with other immune related gene expressions previously identified. Finally, this method could help to classify and help the diagnosis of PitNETs using immune-related gene expression.

Of the 22 types of immune cells estimated by the CIBERSORT algorithm, the most commonly represented cells in our data included M2 macrophages, CD4+ and CD8+ lymphocytes, as well as neutrophils and NK cells. Perhaps the most consistent finding among studies analyzing immune cell infiltration in PitNET is the over-representation of pro-tumorigenic, polarized M2 macrophages [5], while pro-inflammatory M1 macrophages predominate in non-tumoral pituitary glands. Principe et al. studied 28 pitNET by flow cytometry and found a higher percentage of CD68+ and CD163+ macrophages in nonfunctioning gonadotrophinomas than in GH-secreting PitNET, and the presence of these immune cells correlated with invasiveness [15]. In contrast, in a transcriptomic study comprising 210 PitNET with immunohistochemical validation in 77 of these lesions, Luo et al. found that POU1F1-derived neoplasms had a significant increase in M2 macrophages than TBX19- and NR5A1-derived PitNET or non-tumoral pituitaries [16]. In this large cohort, the presence of M2 macrophages was positively associated with tumor volume [16]. Interestingly, a previous study reported that GH-secreting PitNET positive for *AIP* germline mutations had greater amounts of CD68+ macrophage infiltration than sporadic tumors and non-tumoral pituitary glands [17]. Although our data confirms that M2 macrophages are the most common immune cell present in the PitNET microenvironment, we did not find any variations among the different tumor types and their presence was neither associated with invasiveness nor with clinical behavior. Besides M2 macrophages, we found CD4+ and CD8+ lymphocytes, as well as NK cells, infiltrating PitNET as part of their microenvironment, although we could not identify significant associations with the clinical behavior of these lesions. In 1998, Heshmati et al. reported a retrospective series of 1400 patients, whose PitNET were evaluated by immunohistochemistry using specific antibodies against LCA (human common leukocyte antigen), CD45RO, which identifies human T lymphocytes, and CD20, which identifies B lymphocytes [3]. In this large series, 40 tumors had evidence of lymphocytic infiltration, largely T cells: 19 of the 411 prolactinomas, 8 of th 310 multihormonal tumors, 4 of the 137 somatotrophinomas, and 4 of the 44 immunostaining for α-subunit; no correlations with PitNET clinical-biological behavior were sought [3]. Since then, several groups have investigated the prevalence and pathophysiology of lymphocytic infiltration using more sensitive strategies, including flow cytometry, immunofluorescence, and deconvolution analysis based on transcriptomic information. Zhou et al. carried out RNA sequencing of 115 pitNET and computationally inferred the presence of immune cell infiltrates [8]. Functioning PitNET, particularly GH-secreting neoplasms, had significantly more immune infiltrates than nonfunctioning PitNET, B lymphocytes, and, to a lesser extent, CD8+ cytotoxic cells, being the most commonly found cells [8]. Another study found that PitNET with a greater Ki67 proliferative index had lower CD8+/CD4+ and CD8+/FOXP3+ ratios, as well as more infiltrating macrophages, CD4+ helper T cells, and B cells, than tumors with values below 3% [5]. In nonfunctioning PitNET, invasiveness has been associated with higher levels of CD8+ cytotoxic T cells and a higher FOXP3+/CD8+ ratio [7,18,19]. 

The TME of PitNET comprises not only immune cells but also a myriad of cytokines, chemokines, hormones, extracellular matrix proteins, and other relevant molecules that can potentially influence tumor progression and invasion, as well as the response to targeted molecular therapies such as ICi and tyrosine kinase inhibitors. In our study, differential gene expression analysis revealed that genes encoding relevant immune response molecules such as *IL-4I1*, *IL-36*, *IL-17*, and *CCL5* were upregulated in all PitNET. IL4I1 is an enzyme that oxidizes phenylalanine and has been described as a metabolic immune checkpoint that promotes tumor progression. It could enhance tumor development by diminishing CD8+ T cells. It is expressed in hematological malignancies such as B cell lymphoma as well as in solid tumors. This gene could be related to macrophage infiltration in tumors. It has been shown to have immunoregulatory properties and could represent an attractive therapeutic target in several tumors [20,21]. IL-36 plays a role in different cell types as shown in human and animal models via binding to its cognant receptor. Active IL-36 induces the expression of inflammatory cytokines and chemokines from keratinocytes, including IL-17C, granulocyte colony-stimulating factor (G-CSF), IL-8, chemokine ligand 1 (CXCL-1), and CCL-20. IL-36 also induces IL-17A and TNFα expression in human keratinocytes, which could be synergized by IL-22. Interestingly, IL-36 acts synergically with IL-17A and TNFα to increase the expression of IL-6 and IL-8 in primary human keratinocytes. IL-36 is a potent inductor of macrophage, T cell, and neutrophil chemokine synthesis and secretion. It has been found to be expressed in lung, colon, and stomach neoplasms where it contributes to proliferation, invasion, and migration [22,23]. CCL5 and IL17 are also expressed in several solid and hematological malignancies, influencing ECM (extracellular matrix) remodeling and migration, cancer stem cell expansion, DNA damage repair, metabolic reprogramming, and angiogenesis [24,25].

Genes encoding relevant immune response proteins were specifically over expressed in PitNET compared to non-tumoral pituitary gland, according to the PitNET lineage: IL-34 in NR5A1-derived nonfunctioning PitNET, which could promote cell differentiation, proliferation, and survival [26,27]; *IL-20RA* mRNA was up-regulated in TBX19-derived ACTH-secreting PitNET, and could promote tumor initiation, drive metastasis, activate the JAK1-STAT3-SOX2 pathway, increase *PD-L1* expression, and reduce the recruitment of anticancer lymphocytes [28]; *IL-2RB* mRNA was up-regulated in POU1F1-derived lesions and is known to influence malignant behavior. In several tumor types, IL2RB could be used as a potential predictive biomarker [29] of therapy with ICi, particularly in colorectal cancer [29]. The up-regulation of IL-34 acts as a chemoattractant of macrophages [30], whereas IL2RB correlates with T cell infiltration [29]. Marques et al. evaluated the cytokine secretome in primary cultures of 16 nonfunctioning PitNET and 8 somatotrophinomas by means of a high-throughput immunoassay designed to detect 42 different cytokines [7]. In this study, PitNET with a high macrophage and neutrophil content overexpressed *IL-8*, among others, whereas in those with a high CD8+ T lymphocyte content *CCL2, CCL4*, and *VEGF* were upregulated, although this did not significantly correlate with clinical characteristics [7]. Overexpression of *CCL17* by M2 macrophages has been associated with PitNET size and invasiveness, apparently through the activation of the mTOR signaling pathway [31]. Furthermore, invasive GH- and TSH-secreting PitNET were infiltrated predominantly with myeloid cells and genes encoding *CXCL* and *IFITM3* were upregulated.

The response to immunotherapy with ICi depends on the expression of *PD1, PD-L1*, and *CTLA4*, which are in fact the targets of these powerful monoclonal antibodies [32]. In some studies, PitNET progression has been associated with overexpression of *PD1* and *PD-L1* and a greater immune cell infiltration [7,33]. Previous reports have been associated the up-regulation of PD1, PD-L1, and CTLA4 ligands CD80 and CD86 with aggressiveness in proliferative PitNETs. Interestingly, higher expression of these genes is related to higher CD4+, CD8+, and FOXP3+ T cells as well as with higher CD163 macrophages tumor infiltration [34]. It was previously reported that POU1F1 lineage, particularly TSH and GH PitNETs, showed high transcription levels of PD-L1 and might be responsive to immune based therapies, whereas inversely the NR5A1 lineage showed lower levels PD-L1 and might not be as responsive to immune based therapies [35,36]. We observed a more heterogeneous behavior based on the inmunophenoscore results, which evaluated the PD1, PD-L1, and CTLA-4 gene expression without regard to PitNET lineage; as a result, we decided to complement the three gene expression with complementary genes to generate a more robust predictive molecular profiles. In our study, other immunotherapy response genes were found to be overexpressed specifically in pitNET: *CCL18* in ACTH-secreting PitNET, *IL-5RA* in GH-secreting PitNET, and *HLA-B* in nonfunctioning PitNET. Here we provide evidence of molecular profiles that could be used for the benefit of patients who could be recipients of immune based therapies. Despite the success of PD1-PDL1 immune based therapies, only a subset of patients show benefits, and response rates vary widely [37]; therefore, we present a more complete landscape of potentially molecular markers that could help to predict patients with therapeutic response odd in favor and to understand targets that could be associated with extended benefits.

We acknowledge the limitations of our study, particularly those regarding ICi, since there were very few clinical associations due to the small number of aggressive tumors or tumor-specific types in our cohort. Another limitation of our study was the lack of Th1, Th2, or Th17 or other T cell phenotype characterizations. Nevertheless, our data are in line with previously reported studies.

## 5. Conclusions

In conclusion, the microenvironment of PitNET is a complex array of immune cell infiltrates, cytokines, chemokines, and extracellular matrix components. Although M2 macrophages are the most consistently reported immune cells infiltrating PitNET, information regarding the presence of other immune cells and chemical mediators is heterogeneous. Further investigations are required to elucidate how this microenvironment influences the biological behavior of these neoplasms.

## Data Availability

The raw data (CEL files) has been uploaded into the Gene Expression Omnibus (GEO), which is hosted by the National Center for Biotechnology Information (NCBI) under the accession number GSE147786.

## Supplementary Materials

The following supporting information can be downloaded at: https://www.mdpi.com/article/10.3390/genes15050531/s1, Figure S1: Tumor cell components by deconvolution.
