# Supplementary figures and images for: The Immune Microenvironment Landscape of Pituitary NeuroEndocrine Tumors, a Transcriptomic Approach
Source: Genes (Basel). 2024 Apr 24;15(5):531. doi: 10.3390/genes15050531 (PMC11120841; doi:10.3390/genes15050531)

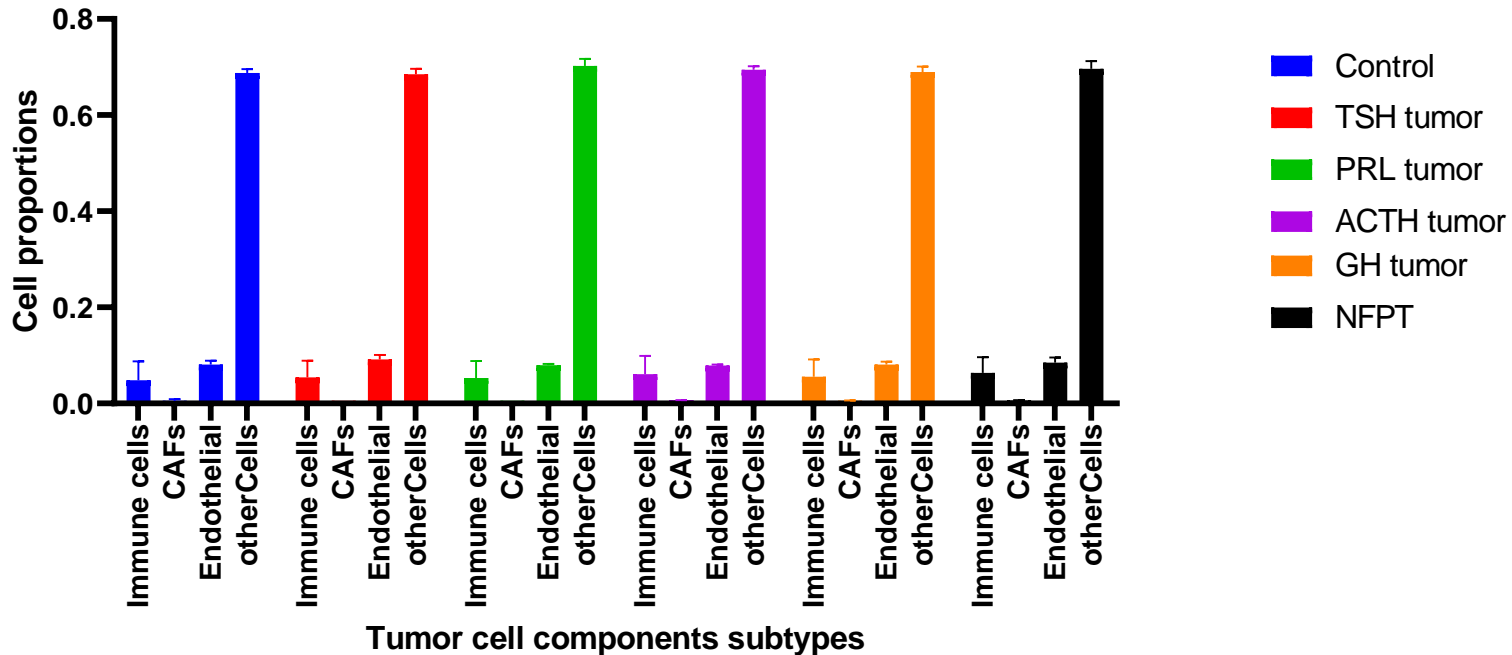

Supplement: Supplementary file 1 [file genes-15-00531-s001.zip › genes-2957598-supplementary.pdf]
